# 2794. Assessing the impact of inappropriate antibiotic therapy on mortality among intensive care unit patients

**DOI:** 10.1093/ofid/ofad500.2405

**Published:** 2023-11-27

**Authors:** Rogério Pereira, Sinval Lins Silva, Braulio Couto

**Affiliations:** Hospital Felício Rocho, Belo Horizonte, Minas Gerais, Brazil; Hospital Felício Rocho, Belo Horizonte, Minas Gerais, Brazil; Biobyte Tecnologia em Epidemiologia, Belo Horizonte, Minas Gerais, Brazil

## Abstract

**Background:**

This study evaluates the potential association between inappropriate antibiotic therapy and adverse clinical outcomes in infected patients admitted to ICUs.

**Methods:**

We analyzed data from infected patients admitted to a medical-surgical Intensive Care Unit (ICU) in Belo Horizonte, a city with a population of three million in Brazil. The data was collected between January 2020 and June 2021, and included patients with healthcare-associated and community-acquired infections. In order to determine the appropriateness of antibiotic therapy, cultures and susceptibility reports were utilized. Empiric antimicrobial therapy was classified as inappropriate if the isolated strain was resistant to all empirically administered antimicrobials, while it was considered adequate if the isolated strain was susceptible to at least one of the antimicrobials used empirically.

**Results:**

A total of 470 patients who received antimicrobials in the ICU were included in the analysis, all of whom had a positive culture. Among them, 65 patients experienced a FAILURE of the antimicrobial regimen, resulting in an overall failure rate of 14%. The monthly rate of inappropriate antibiotic therapy showed significant heterogeneity during the study period, ranging from 100% success in March 2021 to a failure rate of 33% in June 2021 (Fig 1). The failure rate of antimicrobial regimen was found to be significantly higher in cases where the infection was caused by multidrug-resistant (MR) strains compared to multisensitive (MS) strains, as shown in Table 1. Failure of the antimicrobial regimen was a significant risk factor for patient death, regardless of whether the infection was caused by MS or MR strains (Tables 1 and Fig 2). Patients who died tended to receive more antimicrobials compared to those who were discharged, with an average of three antimicrobials prescribed for patients who progressed to death, while discharged patients received an average of two drugs.

**Figure:**
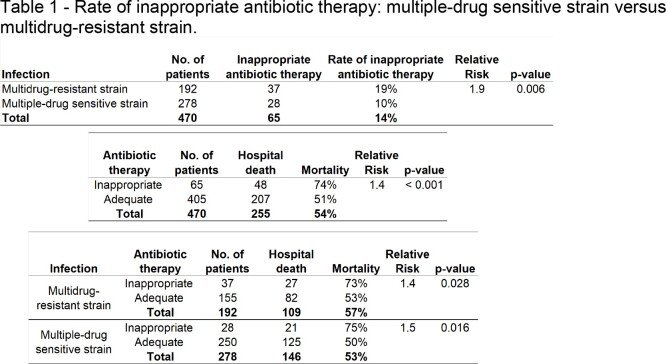

**Figure:**
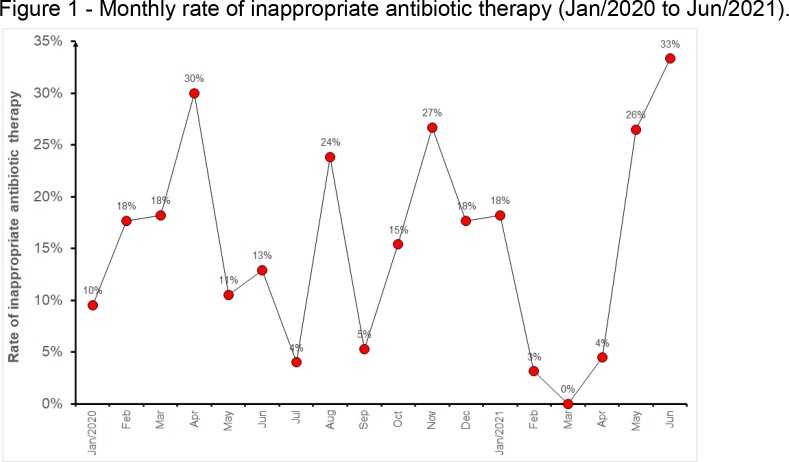

**Figure:**
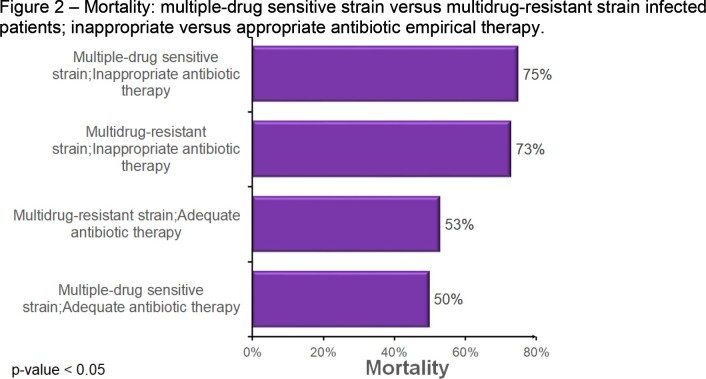

**Conclusion:**

Inappropriate antibiotic therapy in ICU patients is associated with increased mortality risk, regardless of the type of infection. Timely and appropriate antimicrobial therapy is crucial in the management of ICU infections to improve clinical outcomes.

**Disclosures:**

**All Authors**: No reported disclosures

